# Evaluating the Anti-Osteoarthritis Potential of Standardized *Boswellia serrata* Gum Resin Extract in Alleviating Knee Joint Pathology and Inflammation in Osteoarthritis-Induced Models

**DOI:** 10.3390/ijms25063218

**Published:** 2024-03-12

**Authors:** Yean-Jung Choi, Jae In Jung, Jaewoo Bae, Jae Kyoung Lee, Eun Ji Kim

**Affiliations:** 1 Department of Food and Nutrition, Sahmyook University, Seoul 01795, Republic of Korea; yjchoi@syu.ac.kr; 2 Industry Coupled Cooperation Center for Bio Healthcare Materials, Hallym University, Chuncheon 24252, Republic of Korea; jungahoo@hallym.ac.kr; 3 FMCG-Korea Research Institute, FMCG-Korea Co., Ltd., Goyang 10391, Republic of Korea; jerry@fmcg-korea.com (J.B.); ljk1200@fmcg-korea.com (J.K.L.)

**Keywords:** osteoarthritis, *Boswellia serrata*, monosodium iodoacetate, type II collagen, aggrecan, cytokines, matrix metalloproteinase

## Abstract

Osteoarthritis is a widespread chronic degenerative disease marked by the deterioration of articular cartilage, modifications in subchondral bone, and a spectrum of symptoms, including pain, stiffness, and disability. Ultimately, this condition impairs the patient’s quality of life. This study aimed to evaluate the therapeutic efficacy of standardized *Boswellia serrata* gum resin extract (BSRE) in a rat model of monosodium iodoacetate (MIA)-induced osteoarthritis. A total of 60 rats were allocated into six groups: normal control group (NC), osteoarthritis control (injected with MIA, OC), O + B50 (injected with MIA and treated with 50 mg/kg body weight (BW) BSRE), O + B75 (injected with MIA and treated with 75 mg/kg BW BSRE), O + B100 (injected with MIA and treated with 100 mg/kg BW BSRE), and O + M (injected with MIA and treated with 150 mg/kg BW methyl sulfonyl methane). Several parameters, including knee joint swelling, histopathological changes, and the expression of collagen type II alpha 1 (COL2A1) and aggrecan, were comprehensively assessed. Concurrently, the serum levels and mRNA expression of inflammatory mediators, cytokines, and matrix metalloproteinases (MMPs) were analyzed in both the serum and knee joint synovium. The results demonstrated that BSRE significantly mitigated knee joint swelling, cartilage destruction, and tissue deformation. Notably, BSRE administration markedly upregulated the expression of COL2A1 and aggrecan while concurrently reducing levels of nitric oxide, prostaglandin E2, leukotriene B4, interleukin (IL)-6, and tumor necrosis factor (TNF)-α. Furthermore, a substantial decrease was observed in the mRNA expression of inducible nitric oxide synthase, cyclooxygenase-2, 5-lipoxygenase, IL-6, TNF-α and MMP-3 and -13, thereby indicating promising therapeutic implications for osteoarthritis. In conclusion, BSRE exhibited anti-inflammatory properties and inhibited cartilage matrix degradation in a rat model of MIA-induced osteoarthritis, with the O + B100 group showing significant reductions in swelling and notable improvements in joint cartilage damage. These findings illuminate the preventive and therapeutic potential of BSRE for osteoarthritis treatment, emphasizing the criticality of exhaustive evaluation of novel compounds.

## 1. Introduction

Osteoarthritis stands as a quintessential chronic degenerative disease marked by the degradation of articular cartilage, alterations in subchondral bone, and a cascade of symptoms culminating in pain, stiffness, and disability, consequently diminishing the patient’s quality of life [1,2,3]. The multifactorial etiology of osteoarthritis renders it challenging to arrest its progression, necessitating an approach aimed at improving joint function, ameliorating quality of life, and mitigating pain while circumventing adverse effects [4,5].

In the therapeutic landscape, conventional agents, such as painkillers, non-steroidal anti-inflammatory drugs, and corticosteroids, are pivotal for symptomatic relief but are accompanied by potential side effects and toxicity upon prolonged use [6,7,8]. This has kindled a growing interest in health-functional foods, deemed as a safer alternative for preventing and alleviating osteoarthritis symptoms [9,10,11].

*Boswellia serrata* gum resin, an oleo-gum resin derived from the *Boswellia serrata* tree indigenous to the Indian highlands, has historically been used as a remedy for chronic inflammatory diseases and arthritis [12]. It includes six types of boswellic acids, notably, keto-β-boswellic acid (KBA) and 3-O-acetyl-11-keto-β-boswellic acid (AKBA), which exhibit pronounced antioxidant and anti-inflammatory properties [13,14,15]. Previous research has substantiated the anti-osteoarthritis efficacy of FJH-UBS, a derivative of *Boswellia serrata* gum resin enriched in KBA and AKBA, in interleukin (IL)-1β-stimulated human SW1353 chondrocytes [16] and osteoarthritis-induced animal models of osteoarthritis [17].

Amidst the established efficacy of *Boswellia serrata* gum resin in ameliorating osteoarthritis, a plethora of its extracts are being utilized around the world as health-functional ingredients [18,19,20,21,22]. Nonetheless, the inherent variability in the composition of natural extracts, influenced by the origin of raw materials and extraction methodologies, necessitates rigorous verification of the safety and efficacy of each *Boswellia serrata* gum resin extract prior to its application to the human body.

The focal point of this study is standardized *Boswellia serrata* gum resin extract (BSRE, FlexiBos^®^). The intent is to garner scientific evidence through animal application testing, thus substantiating the potential of BSRE as a functional material to improve osteoarthritis. This endeavor seeks to contribute new insights into the therapeutic potential of BSRE, addressing a critical gap in the existing body of research and underscoring the necessity for meticulous evaluation of the safety and efficacy of such novel compositions in the context of osteoarthritis treatment [23,24]. To carry this out, we attempted to investigate the anti-osteoarthritic effect of BSRE in monosodium iodoacetate (MIA)-induced osteoarthritis Sprague–Dawley (SD) rats. Possible mechanisms underlying this effect were also explored.

## 2. Results

### 2.1. BSRE Alleviates Knee Joint Swelling in MIA-Induced Osteoarthritis Rats

**Figure 1:**
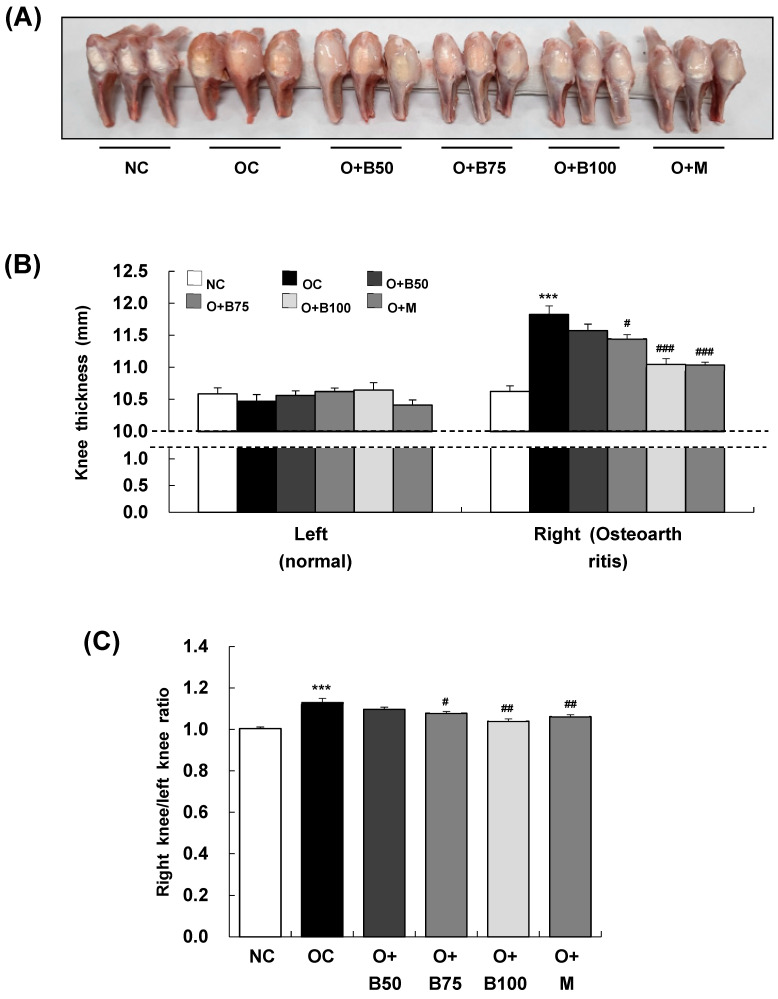
Effect of BSRE on knee thickness in SD rats with MIA-induced osteoarthritis. SD rats were orally administered either BSRE (50, 75, 100 mg/kg BW/day) or MSM (150 mg/kg BW/day) for 5 weeks. After 14 days of administration of BSRE or MSM, MIA was injected into the intra-articular joint of the right knee. We conducted the measurements and sample harvesting 3 weeks post-MIA injection, specifically, on day 21, to assess the effects of the treatments. (**A**) Representative images of right knees (*n* = 10). (**B**) The thickness of left (normal) and right (osteoarthritic) knees was measured and (**C**) the ratio of right/left knee thickness was calculated. Each bar represents the mean ± SEM (*n* = 10). The significance of differences was evaluated using Student’s *t*-test and one-way analysis of variance (ANOVA). *** *p* < 0.001: significantly different from the NC group. ^#^ *p* < 0.05, ^##^ *p* < 0.01, ^###^ *p* < 0.001: significantly different from the OC group.

To assess the impact of BSRE on osteoarthritis-induced knee joint swelling, we conducted measurements and sample harvesting 3 weeks post-MIA injection, specifically, on day 21, measuring the thickness of both the right knee joint, where osteoarthritis was induced by MIA injection, and the left knee joint, which remained un-injected, as depicted in Figure 1A. There was no significant difference in the thickness of the left knee joint across all test groups (Figure 1B). However, a notable increase in the thickness of the right knee joint was observed in the osteoarthritis control group (OC) compared to the normal control group (NC) (*p* < 0.001). Additionally, a significant reduction in thickness was recorded in the osteoarthritis + 75 mg/kg body weight (BW)/day BSRE-treated group (O + B75), osteoarthritis + 100 mg/kg BW/day BSRE-treated group (O + B100), and osteoarthritis + 150 mg/kg BW/day methyl sulfonyl methane (MSM)-treated group (O + M) compared to the OC group (*p* < 0.05). The ratio of right knee joint thickness to left knee joint thickness, as shown in Figure 1C, was significantly higher in the OC group than in the NC group (*p* < 0.001). This ratio was notably lower in the O + B75, O + B100, and O + M groups compared to the OC group (*p* < 0.05).

### 2.2. BSRE Reduces Cortical Bone Erosions in Rats with MIA-Induced Osteoarthritis

**Figure 2:**
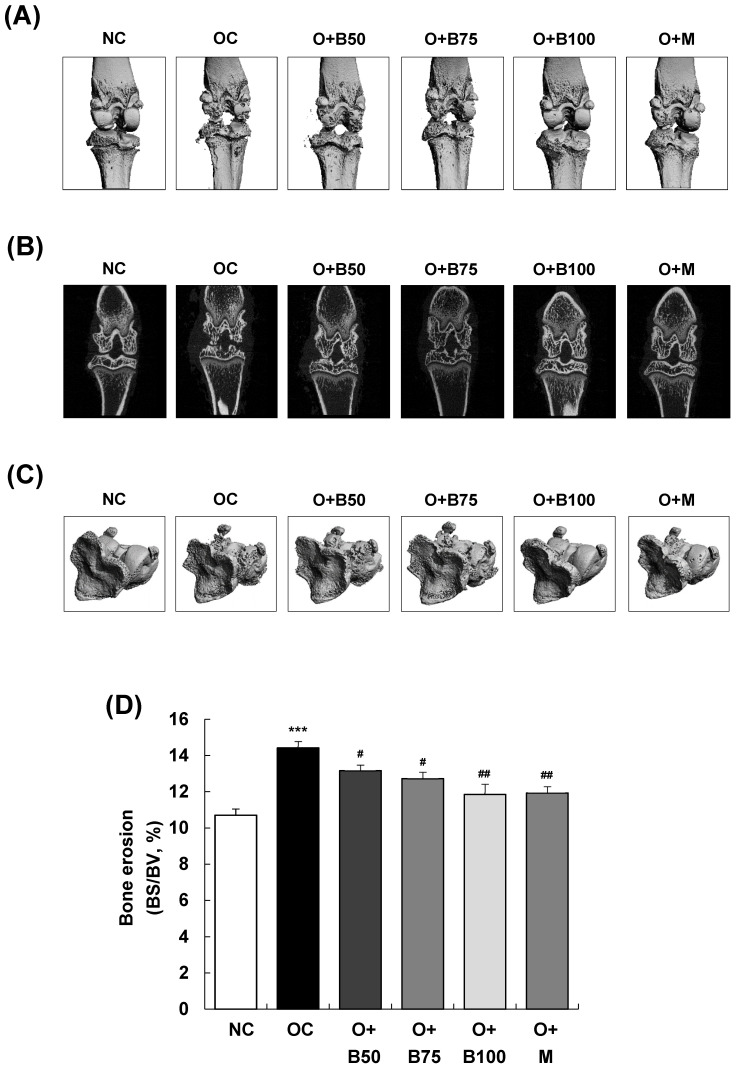

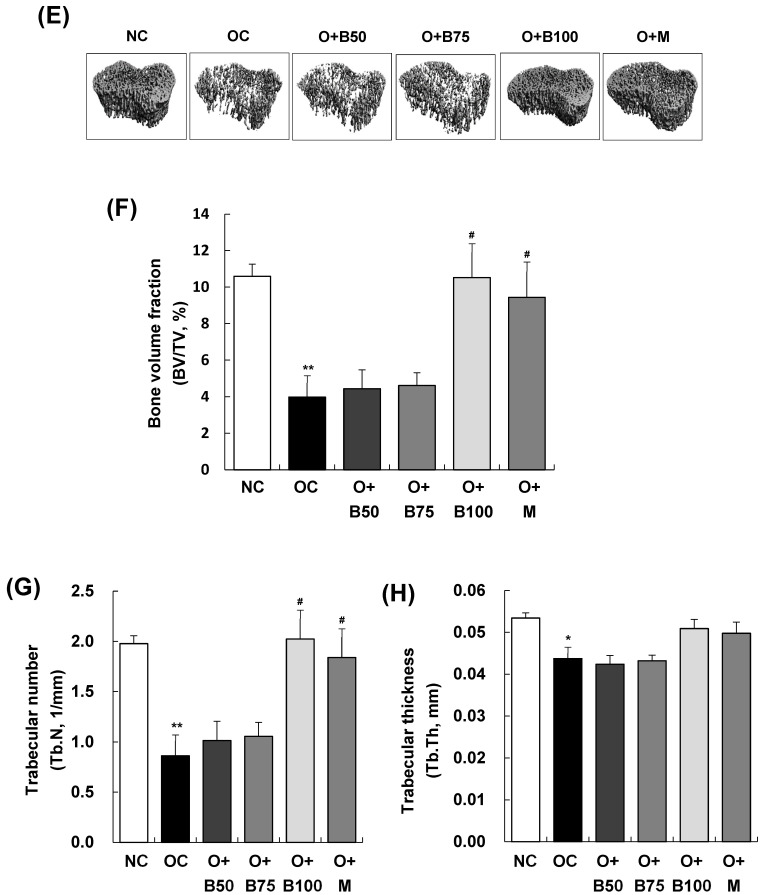
Effect of BSRE on cartilage degradation in SD rats with MIA-induced osteoarthritis. SD rats were administered BSRE and injected with MIA, as described in Figure 1. We conducted the measurements and sample harvesting 3 weeks post-MIA injection—specifically, on day 21—to assess the effects of the treatments. The femorotibial joint was analyzed using micro-CT. (**A**) Three-dimensional micro-CT images (*n* = 5). (**B**) Two-dimensional micro-CT images (*n* = 5). (**C**) Grayscale reconstructed images of the subchondral bone in the femorotibial joint (*n* = 5). (**D**) BS/BV of the subchondral bone in the femorotibial joint (*n* = 5). (**E**) Grayscale reconstructed images of metaphysis in the tibia. (**F**) BV/TV, (**G**) Tb.N, and (**H**) Tb.Th of metaphysis in the tibia (*n* = 5). Each bar represents the mean ± SEM (*n* = 5). The significance of differences was evaluated using Student’s *t*-test and one-way analysis of variance (ANOVA). * *p* < 0.05, ** *p* < 0.01, *** *p* < 0.001: significantly different from the NC group. ^#^ *p* < 0.05, ^##^ *p* < 0.01: significantly different from the OC group.

Micro-computed tomography (micro-CT) analysis conducted 3 weeks post-MIA injection—specifically, on day 21—was utilized to investigate the effect of BSRE on the femorotibial joint, particularly focusing on the epiphysis of the proximal tibia. Significant knee joint damage was observed in the OC group compared to the NC group, as indicated in Figure 2A,B. However, BSRE (osteoarthritis + 50 mg/kg BW/day BSRE-treated group (O + B50), O + B75 group, and O + B100 group) and MSM (O + M group) significantly mitigated the MIA-induced knee joint damage. In Figure 2C,D, the bone surface/bone volume ratio (BS/BV, %) of the subchondral bone, representing the degree of knee joint area erosion, was significantly higher in the OC group than in the NC group (*p* < 0.001). Nonetheless, a significant reduction in the BS/BV ratio was observed in the O + B50, O + B75, O + B100, and O + M groups compared to the OC group (*p* < 0.05).

Figure 2E–H depict that the bone volume fraction (bone volume/total volume ratio, BV/TV, %), representing the proportion of cancellous bone within the volume of interest; the trabecular number (Tb.N, 1/mm), indicating the number of cancellous bones per unit length within the volume of interest; and trabecular thickness (Tb.Th, mm) were significantly lower in the OC group than in the NC group (*p* < 0.001). Notably, in the O + B100 and O + M groups, these parameters did not decrease as in the OC group but remained significantly higher, indicating a protective effect against osteoarthritis-induced bone structure changes (*p* < 0.05).

### 2.3. BSRE Alleviates Histomorphological Changes in Knee Joint Cartilage in a Rat Model of MIA-Induced Osteoarthritis

**Figure 3:**
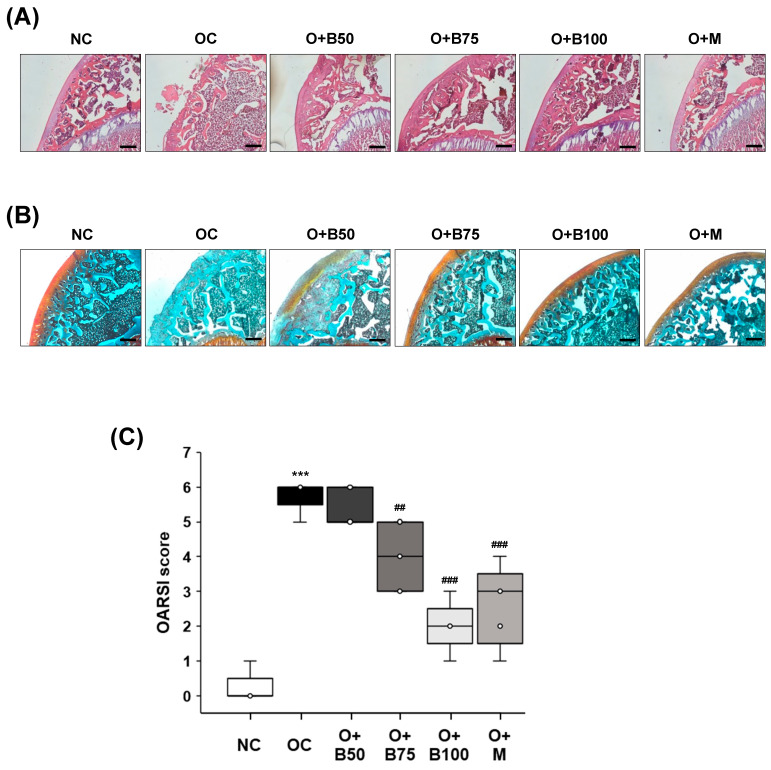
Effect of BSRE on histological changes in the articular cartilage of SD rats with MIA-induced osteoarthritis. SD rats were administered BSRE and injected with MIA, as described in Figure 1. We conducted the measurements and sample harvesting 3 weeks post-MIA injection, specifically, on day 21, to assess the effects of the treatments. Articular cartilage was stained with (**A**) H&E and (**B**) safranin O and subjected to Osteoarthritis Research Society International (OARSI) scoring. Representative staining images are shown (*n* = 5). Magnification, 100×. (**C**) OARSI was scored (*n* = 5). Each bar represents the mean ± SEM (*n* = 5). The significance of differences was evaluated using Student’s *t*-test and one-way analysis of variance (ANOVA). *** *p* < 0.001: significantly different from the NC group. ^##^ *p* < 0.01, ^###^ *p* < 0.001: significantly different from the OC group.

Histomorphological changes in knee joint cartilage were observed through hematoxylin and eosin (H&E) staining, conducted 3 weeks post-MIA injection—specifically, on day 21—as depicted in Figure 3A. The articular cartilage tissue in the NC group exhibited smoothness and preservation with no evident deformation or damage. In contrast, the OC group displayed deformed and damaged cartilage tissue with a worn surface. The administration of BSRE and MSM, notably, in the O + B75, O + B100, and O + M groups, mitigated the cartilage damage, bringing improvement and producing levels comparable to the NC group.

Proteoglycan in cartilage tissue was observed using Safranin O staining, the results of which, conducted 3 weeks post-MIA injection—specifically, on day 21—are presented in Figure 3B. In the NC group, cartilage tissue remained in a normal state, with uniform proteoglycan distribution and no tissue deformation or loss. Conversely, the OC group showed severely damaged cartilage tissue and a considerable loss of proteoglycan, which was absent from the cartilage surface. The O + B75, O + B100, and O + M groups exhibited alleviated cartilage damage and reduced proteoglycan loss compared to the OC group, with the O + B75 and O + B100 groups showing more pronounced effects than the O + B50 group.

Osteoarthritis Research Society International (OARSI) score, representing the severity of knee osteoarthritis, was markedly higher in the OC group than in the NC group (*p* < 0.001). Compared to the OC group, OARSI scores were significantly reduced in the O + B75, O + B100, and O + M groups (Figure 3C), with these measurements conducted 3 weeks post-MIA injection, specifically, on day 21.

### 2.4. BSRE Alleviates COL2A1 and Aggrecan Loss in Articular Cartilage

**Figure 4:**
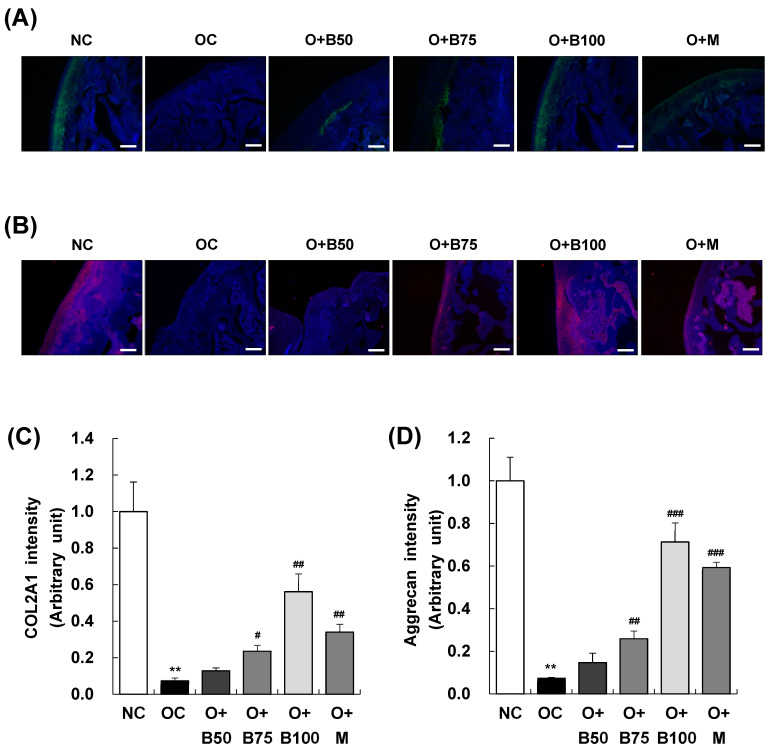
Effect of BSRE on the expression of COL2A1 and aggrecan in the articular cartilage of SD rats with MIA-induced osteoarthritis. SD rats were administered BSRE and injected with MIA, as described in Figure 1. We conducted the measurements and sample harvesting 3 weeks post-MIA injection, specifically on day 21, to assess the effects of the treatments. Articular cartilage was stained with (**A**) COL2A1 and (**B**) aggrecan antibodies. Representative staining images are shown (*n* = 5). Magnification, 100×. (**C**,**D**) Staining intensity of the indicated proteins was quantified. Each bar represents the mean ± SEM (*n* = 5). The significance of differences was evaluated using Student’s *t*-test and one-way analysis of variance (ANOVA). ** p < 0.01: significantly different from the NC group. ^#^ *p* < 0.05, ^##^ *p* < 0.01, ^###^ *p* < 0.001: significantly different from the OC group.

Immunofluorescence (IF) staining conducted 3 weeks post-MIA injection, specifically, on day 21, was utilized to investigate the expression of collagen type II alpha 1 (COL2A1) and aggrecan in articular cartilage tissue. The findings, illustrated in Figure 4A,B revealed a significant decrease in COL2A1 and aggrecan expression in the OC group compared to the NC group. In contrast, the O + B75, O + B100, and O + M groups exhibited relatively higher expressions of COL2A1 and aggrecan compared to the OC group, indicating a less pronounced downregulation due to OA (Figure 4C,D).

### 2.5. BSRE Decreases the Content of Inflammatory Mediators and Inflammatory Cytokines in Serum

**Figure 5:**
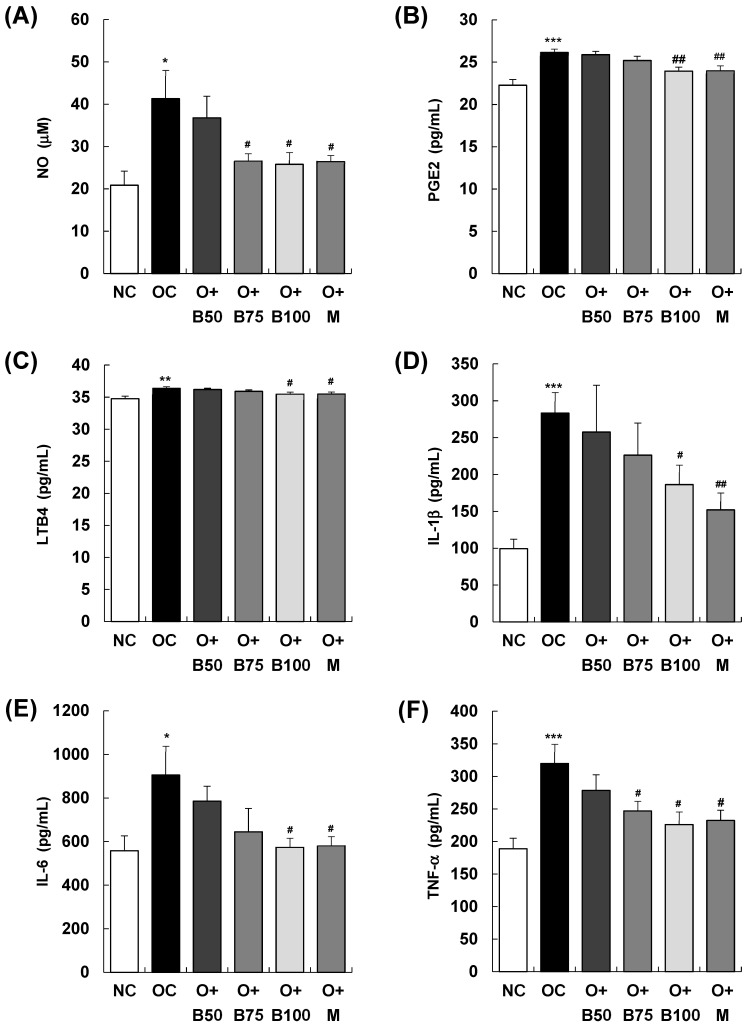
Effect of BSRE on the levels of inflammatory mediators and cytokines in the serum of SD rats with MIA-induced osteoarthritis. SD rats were administered BSRE and injected with MIA, as described in Figure 1. We conducted the measurements and sample harvesting 3 weeks post-MIA injection, specifically, on day 21, to assess the effects of the treatments. Blood was collected and the serum was prepared. The serum levels of (**A**) NO, (**B**) PGE2, (**C**) LTB4, (**D**) IL-1β, (**E**) IL-6, and (**F**) TNF-α were measured with the relevant kits. Each bar represents the mean ± SEM (*n* = 5). The significance of differences was evaluated using Student’s *t*-test and one-way analysis of variance (ANOVA). * *p* < 0.05, ** *p* < 0.01, *** *p* < 0.001: significantly different from the NC group. ^#^ *p* < 0.05, ^##^ *p* < 0.01: significantly different from the OC group.

As illustrated in Figure 5, the serum concentrations of the inflammatory mediators nitric oxide (NO), prostaglandin E2 (PGE2), and leukotriene B4 (LTB4) exhibited a significant increase in the OC group compared to the NC group (*p* < 0.001), with measurements and sample harvesting conducted 3 weeks post-MIA injection, specifically, on day 21. In particular, Figure 5A depicts a notable reduction in serum NO content in the O + B75, O + B100, and O + M groups compared to the OC group. Similarly, Figure 5B,C illustrate significant decreases in serum PGE2 and LTB4 contents in the O + B100 and O + M groups compared to the OC group (*p* < 0.05). Moreover, there was a significant elevation in the serum content of the inflammatory cytokines interleukin (IL)-1β, IL-6, and tumor necrosis factor (TNF)-α in the OC group relative to the NC group (*p* < 0.001, *p* < 0.05, *p* < 0.001, respectively). As depicted in Figure 5D, serum IL-1β content was significantly reduced only in the O + M group compared to the OC group (*p* < 0.05), while Figure 5E,F demonstrate significant reductions in serum IL-6 and TNF-α contents in the O + B75, O + B100, and O + M groups relative to the OC group (*p* < 0.05), aligning with the timing of the post-MIA injection assessments.

### 2.6. BSRE Reduces MMP-3 and MMP-13 Production in Serum

**Figure 6:**
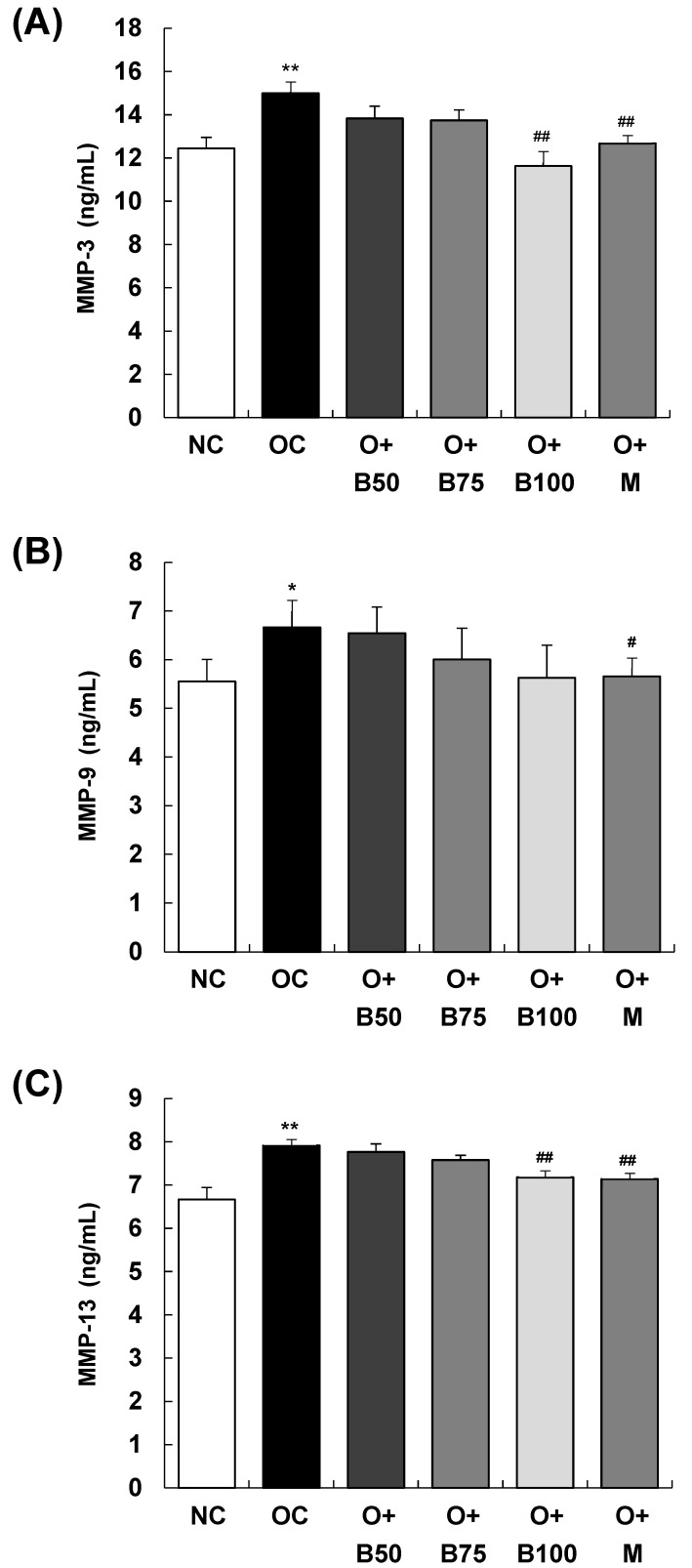
Effect of BSRE on the levels of MMPs in the serum of SD rats with MIA-induced osteoarthritis. SD rats were administered BSRE and injected with MIA, as described in Figure 1. We conducted the measurements and sample harvesting 3 weeks post-MIA injection, specifically, on day 21, to assess the effects of the treatments. Blood was collected and the serum was prepared. The serum levels of (**A**) MMP-3, (**B**) MMP-9, and (**C**) MMP-13 were measured with the relevant ELISA kits. Each bar represents the mean ± SEM (*n* = 5). The significance of differences was evaluated using Student’s *t*-test and one-way analysis of variance (ANOVA). * *p* < 0.05, ** *p* < 0.01: significantly different from the NC group. ^#^ *p* < 0.05, ^##^ *p* < 0.01: significantly different from the OC group.

The serum concentrations of matrix metalloproteinase (MMP)-3, MMP-9, and MMP-13, measured 3 weeks post-MIA injection—specifically, on day 21—are presented in Figure 6. The OC group exhibited significantly elevated concentrations of MMP-3, MMP-9, and MMP-13 compared to the NC group (*p* < 0.01, *p* < 0.05, *p* < 0.01, respectively). Figure 6A,C show that the concentrations of MMP-3 and MMP-13 were significantly diminished in the O + B100 and O + M groups (*p* < 0.05). However, the administration of BSRE did not affect the MMP-9 content in serum in the osteoarthritis test group, as seen in Figure 6B.

### 2.7. BSRE Suppresses mRNA Expression of Inflammatory Mediators and Inflammatory Cyto-Kines in Knee Joint Synovium

**Figure 7:**
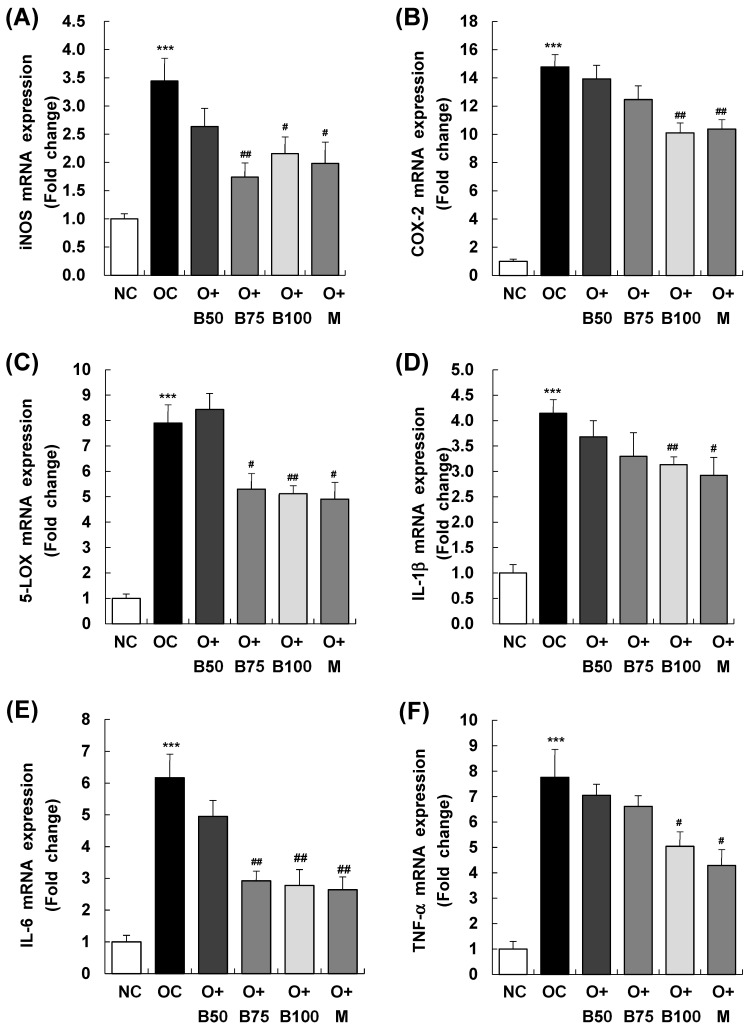
Effect of BSRE on mRNA expression of inflammatory mediators and cytokines in the synovia of SD rats with MIA-induced osteoarthritis. SD rats were administered BSRE and injected with MIA, as described in Figure 1. We conducted the measurements and sample harvesting 3 weeks post-MIA injection, specifically, on day 21, to assess the effects of the treatments. Total RNA in the synovia was extracted and reverse transcribed, and real-time PCR was performed. (**A**) iNOS, (**B**) COX-2, (**C**) 5-LOX, (**D**) IL-1β, (**E**) IL-6, and (**F**) TNF-α mRNA expression was normalized to that of GAPDH and represented as relative to that of the NC group. Each bar represents the mean ± SEM (*n* = 5). The significance of differences was evaluated using Student’s *t*-test and one-way analysis of variance (ANOVA). *** *p* < 0.001: significantly different from the NC group. ^#^ *p* < 0.05, ^##^ *p* < 0.01: significantly different from the OC group.

Figure 7 presents the results of mRNA expression analysis for inducible nitric oxide synthase (iNOS), cyclooxygenase-2 (COX-2), and 5-lipoxygenase (5-LOX)—enzymes responsible for the synthesis of inflammatory mediators NO, PGE2, and LTB4, respectively. These measurements and sample harvestings were conducted 3 weeks post-MIA injection, specifically, on day 21, after extracting total RNA from the knee joint synovium and conducting real-time reverse transcription polymerase chain reaction (RT-PCR). Figure 7A reveals that the OC group had a significant upregulation in iNOS mRNA expression relative to the NC group (*p* < 0.001). In contrast, iNOS mRNA levels were markedly reduced in the O + B75, O + B100, and O + M groups compared to the OC group (*p* < 0.05).

Similarly, as displayed in Figure 7B,C, COX-2 and 5-LOX mRNA levels were notably higher in the OC group than in the NC group (*p* < 0.001). However, the osteoarthritis test groups showed significant reductions in COX-2 mRNA expression in the O + B100 and O + M groups and in 5-LOX mRNA expression in the O + B75, O + B100, and O + M groups, all in comparison to the OC group (*p* < 0.05).

As delineated in Figure 7D–F, mRNA expression of IL-1β, IL-6, and TNF-α was notably elevated in the OC group relative to the NC group. Among the osteoarthritis test groups, only the O + M group exhibited a notable reduction in IL-1β mRNA expression relative to the OC group. IL-6 mRNA levels were significantly reduced in the O + B75, O + B100, and O + M groups, and TNF-α mRNA expression decreased significantly in the O + B100 and O + M groups when compared to the OC group (*p* < 0.05).

### 2.8. BSRE Suppresses MMPs mRNA Expression in Knee Joint Synovium

**Figure 8:**
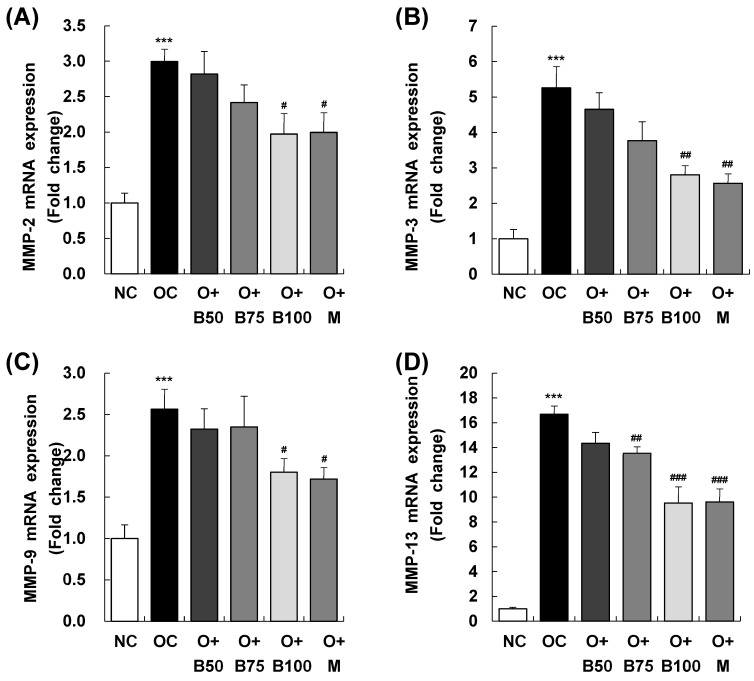
Effect of BSRE on mRNA expression of MMPs in the synovia of SD rats with MIA-induced osteoarthritis. SD rats were administered BSRE and injected with MIA, as described in Figure 1. We conducted the measurements and sample harvesting 3 weeks post-MIA injection, specifically, on day 21, to assess the effects of the treatments. Total RNA in the synovia was extracted, reverse transcribed, and real-time PCR was performed. (**A**) MMP-2, (**B**) MMP-3, (**C**) MMP-9, and (**D**) MMP-13 mRNA expression was normalized to that of GAPDH and represented as relative to that of the NC group. Each bar represents the mean ± SEM (*n* = 5). The significance of differences was evaluated using Student’s *t*-test and one-way analysis of variance (ANOVA). *** *p* < 0.001: significantly different from the NC group. ^#^ *p* < 0.05, ^##^ *p* < 0.01, ^###^ *p* < 0.001: significantly different from the OC group.

Figure 8 elucidates the results of MMP-2, MMP-3, MMP-9, and MMP-13 mRNA expression in the knee joint synovium, with measurements and sample harvesting conducted 3 weeks post-MIA injection, specifically, on day 21, to assess the effects of the treatments. Each of these expressions was notably upregulated in the OC group compared to the NC group. Within the osteoarthritis test groups, the O + B100 and O + M groups saw a marked reduction in MMP-2 mRNA levels compared to the OC group. MMP-3 mRNA levels dropped significantly in the O + B75, O + B100, and O + M groups, while MMP-9 mRNA expression decreased notably only in the O + M group. The mRNA expression of MMP-13 was significantly reduced in the O + B75, O + B100, and O + M groups compared to the OC group (*p* < 0.05).

## 3. Discussion

The results of this study yielded several key findings. Firstly, it was observed that the OC group manifested a notable increase in knee joint swelling and cartilage damage in comparison to the NC group. Secondly, the administration of BSRE led to a significant diminution in knee joint swelling and effectively mitigated the cartilage destruction induced by MIA. Notably, higher doses of BSRE showcased more pronounced effects. Thirdly, a histomorphological examination indicated that BSRE played a crucial role in alleviating the deformation and damage seen in the cartilage tissue in the OC group, thus enhancing the cartilage health to levels akin to those in the NC group. Fourthly, the OC group demonstrated a significant reduction in the expression of the essential cartilage proteins COL2A1 and aggrecan compared to the NC group. Notably, the groups treated with BSRE did not exhibit such pronounced downregulation, with their levels of COL2A1 and aggrecan expression being significantly greater than those in the OC group. Furthermore, the OC group exhibited elevated levels of inflammatory mediators (NO, PGE2, LTB4) and cytokines (IL-1β, IL-6, TNF-α) in the serum. These levels were significantly abated by BSRE, indicating an attenuation in inflammation. Additionally, the study revealed that the OC group had elevated serum concentrations of MMP-3, MMP-9, and MMP-13. Remarkably, these were significantly reduced by BSRE, with the exception of MMP-9, which BSRE did not affect. Moreover, the mRNA expression levels of inflammatory mediators, cytokines, and MMPs in the knee joint synovium were notably elevated in the OC group but underwent a significant reduction in the groups treated with BSRE. Collectively, these findings underscore the potential preventive and therapeutic efficacy of BSRE in alleviating osteoarthritis-induced knee joint issues, cartilage damage, and inflammation.

Various studies have reported the beneficial impact of *Boswellia serrata* gum resin in ameliorating osteoarthritis [5,6,18,19,20,21,22]. In previous research, particularly highlighted in Jung et al., 2023 [17], *Boswellia serrata* gum resin extract (FJH-UBS) was investigated for its therapeutic effects in MIA-induced osteoarthritic Sprague–Dawley rats. Their study, which administered the extract at a dose of 80 mg/kg BW for a duration of 5 weeks, reported notable reductions in knee joint swelling, cartilage loss, and an increased expression of collagen and aggrecan in the cartilage tissue. Building upon these findings, our current study explores the effects of a different *Boswellia serrata* gum resin extract (BSRE—FlexiBos), notable for its higher content of AKBA and KBA (110 mg/g). We evaluated the impact of administering BSRE at dosages ranging from 50 to 100 mg/kg BW over a period of 5 weeks. Our results demonstrate a significant decrease in knee joint swelling and intra-articular cartilage loss; this decrease was particularly pronounced in the group receiving 100 mg/kg BW. Additionally, we observed an upregulation in collagen and aggrecan expression and a concomitant decrease in the expression of inflammatory mediators, cytokines, and MMPs in cartilage tissue. These findings underscore the variability in the efficacy and mechanisms of action of *Boswellia serrata* gum resin extracts, which can be attributed to differences in their origin and extraction processes [6,18].

Osteoarthritis primarily stems from inflammation within the joint, triggered by various inflammatory mediators and cytokines [25]. Patients with osteoarthritis exhibit higher levels of inflammatory cytokines IL-1β, IL-6, and TNF-α in the blood, synovial fluid, and cartilage tissue compared to healthy individuals [26]. Elevated levels of these cytokines in articular cartilage are known to induce cartilage degeneration by promoting the breakdown of the cartilage matrix [27]. Moreover, IL-1β increases the expression of iNOS and COX-2 in cartilage [28,29], leading to the production of NO and PGE2 in chondrocytes, which are associated with the degradation of ECM and the onset of osteoarthritis [30,31]. A previous study on SW1353 cells, human-derived chondrocytes, showed that FJH-UBS reduced the expression of iNOS, PGE2, IL-6, and TNF-α mRNA elevated by IL-1β and influenced the activities of MAPK, NF-κB, and IκBα [16]. In the present study, administering BSRE at doses above 75 mg/kg hindered the progression of osteoarthritis. It led to reduced serum levels of NO, PGE2, LTB4, IL-6, and TNF-α and decreased iNOS, COX-2, and 5-LOX mRNA expression in the cartilage synovium. These findings suggest that BSRE mitigates osteoarthritis by attenuating the inflammatory response. It is important to note that in our study, the inhibition of osteoarthritis progression was observed with pretreatment using BSRE, which is a critical point for considering its translational relevance in future therapeutic applications.

In our MIA-induced osteoarthritis animal model, BSRE administration diminished the production and content of MMP-3 and -13 in the serum and suppressed the expression of MMP-2, -3, and -13 mRNA in the synovium. MMPs play a critical role in various processes, including tissue differentiation, wound healing, organ formation, angiogenesis, and tissue resorption and remodeling [32]. They are proteolytic enzymes that degrade components of bone and cartilage matrix, contributing to joint and cartilage degeneration, and, subsequently, to osteoarthritis. Among various MMPs, MMP-1, MMP-3, and MMP-13 are reported to demonstrate increased expression in osteoarthritis and are closely associated with its development [33]. IL-1β, central to the inflammatory response in osteoarthritis, elevates the expression of MMPs in chondrocytes [16,34], and an increase in MMPs due to elevated IL-1β is identified as one of the primary mechanisms of osteoarthritis [35]. MMPs not only degrade cartilage matrix components but also inhibit their synthesis. Previous studies have shown that MMP-13 impedes the synthesis of type II collagen and aggrecan, the main components of cartilage [36], and that elevated expression of MMPs and reduced expression of type II collagen and aggrecan occur when chondrocytes are stimulated with IL-1β [16,34]. Research by Jung et al. [16] revealed that FJH-UBS inhibits cartilage matrix degeneration by suppressing the expression of MMP-1, MMP-3, and MMP-13 induced by IL-1β in SW1353 cells and increases the expression of aggrecan and type II collagen, which are decreased by IL-1β. In this study, we observed that BSRE treatment resulted in significantly higher expressions of COL2A1 and aggrecan compared to the OC group. This finding suggests that BSRE may contribute to osteoarthritis alleviation by lessening the reduction in these essential cartilage proteins, likely through the suppression of MMPs’ expression, thereby potentially inhibiting the degradation of cartilage ECM. Future research will focus on analyzing the protein expression of MMP-2, MMP-3, MMP-9, and MMP-13 to more fully elucidate the molecular mechanisms by which BSRE impacts osteoarthritis progression, providing valuable insights for developing more effective therapeutic strategies.

The inflammatory response and ECM degradation process in osteoarthritis are regulated through various signaling systems, notably, the MAPK and NF-κB pathways [37,38]. Activation of the MAPK pathway in cartilage increases the expression of inflammatory mediators and MMPs, leading to osteoarthritis [39,40,41]. Similarly, activation of the NF-κB pathway enhances the transcription of inflammatory mediators, cytokines [42], and ECM decomposition factors, contributing to cartilage degradation [29]. Hence, inhibiting the activity of the MAPK and NF-κB signaling systems could be a pivotal target for preventing, mitigating, and treating osteoarthritis. A preceding study found that FJH-UBS suppressed inflammatory response and cartilage degradation by inhibiting the activity of the MAPK and NF-κB signaling systems in an osteoarthritis cell model induced by treating SW1353 cells with IL-1β [16]. Although this study did not investigate the effect of BSRE on these signaling systems, future research into the role of the MAPK and NF-κB pathways in the anti-osteoarthritic effect of BSRE is deemed necessary.

Currently, various forms of Boswellia and Boswellia extracts are utilized to ameliorate osteoarthritis [9,19,20,22], but there is a scarcity of scientific evidence supporting the anti-osteoarthritic efficacy of each product [21,43]. This study demonstrated that BSRE exhibits anti-osteoarthritic efficacy by suppressing inflammatory response and cartilage matrix degradation in an MIA-induced osteoarthritis animal model. While BSRE has shown effective anti-osteoarthritic efficacy at the animal level, human application tests are essential for future work to validate the anti-osteoarthritic efficacy of BSRE in humans, thereby facilitating its use for enhancing joint and cartilage health.

## 4. Materials and Methods

### 4.1. Materials

This study utilized the following materials obtained from respective suppliers: MIA, H&E, and 4′,6-diamidino-2-phenylindole (DAPI) from Sigma-Aldrich Co. (St. Louis, MO, USA); enzyme-linked immunosorbent assay (ELISA) kits for PGE2, LTB4, TNF-α, IL-1β, IL-6, and MMP-9 from R&D Systems (Minneapolis, MN, USA); ELISA kits for MMP-3 and MMP-1 from MyBiosource (San Diego, CA, USA); Griess reagent system from Promega (Madison, WI, USA); fluorochrome-conjugated secondary antibodies (Alexa-488 and 564) from Thermo Fisher Scientific (Waltham, MA, USA); Safranin O from ScienCell Research Laboratories (Carlsbad, CA, USA); anti-aggrecan and anti-COL2A1 antibody from Santa Cruz Biotechnology (Santa Cruz, CA, USA); TRIzol reagent from Invitrogen Life Technologies (Carlsbad, CA, USA); HyperScriptTM RT master mix from GeneAll Biotechnology (Seoul, Republic of Korea); QuantiNova SYBR Green PCR kit from Qiagen (Valencia, CA, USA).

### 4.2. Preparation of BSRE

BSRE (FlexiBos^®^) was generously provided by FMCG-Korea Co., Ltd. (Goyang, Republic of Korea). In a brief overview of the extraction process, *Boswellia serrata* gum resin was extracted with 95% ethanol at 60 °C for 6 h. The extract was filtered and concentrated by vacuum evaporation. To remove lipids, an equal volume of hexane was added to the concentrate; subsequently, the mixture was allowed to stand for 10 min. Then, it was filtered. The filtrate was dried using a vacuum dryer so that the moisture content was less than 5%; maltodextrin (10%) was added, and the mixture was mixed homogenously. The resulting powder was used as BSRE. The sum of AKBA and KBA, a bioactive and indicative compound, was 110 mg/g in BSRE.

### 4.3. Ethical Statement and Animal Handling

The animal experimental protocols adhered to in this study received approval from the Institutional Animal Care and Use Committee of Hallym University (approval number: Hallym 2022-66). All animal experiments were conducted in accordance with the guidelines for care and use of laboratory animals.

Specific pathogen-free male SD rats, aged 6 weeks, were procured from Dooyeol Biotech Co., Ltd. (Seoul, Republic of Korea) and housed at the animal facility of Hallym University, which maintained 23 ± 3 °C and 50 ± 10% relative humidity, with a 12 h light/dark cycle. The rats had ad libitum access to a commercial non-purified rodent diet and tap water.

### 4.4. Experimental Design, Treatment, and Induction of Osteoarthritis

After acclimation for 1 week, the SD rats were randomly assorted into six groups (*n* = 10 rats/group) as follows: (1) normal control group (NC), (2) osteoarthritis control group (OC), (3) osteoarthritis + 50 mg/kg BW/day BSRE-treated group (O + B50), (4) osteoarthritis + 75 mg/kg BW/day BSRE-treated group (O + B75), (5) osteoarthritis + 100 mg/kg BW/day BSRE-treated group (O + B100), (6) osteoarthritis + 150 mg/kg BW/day MSM-treated group (O + M). The rats in each group received oral administration of either BSRE or MSM (Methylsulfonylmethane-positive control), which was dissolved in sterile water, daily for five weeks. BSRE was administered once daily to ensure consistent dosing and experimental conditions. We selected MSM as the positive control for our study due to its established effectiveness in mitigating inflammation and cartilage degeneration, hallmarks of osteoarthritis [44]. This choice enables a pertinent comparison with BSRE, as MSM’s recognized role in osteoarthritis research serves as a valuable benchmark for evaluating the therapeutic potential of BSRE in similar contexts. The rats in the NC and OC groups were administered an equal volume of sterile water as a vehicle by oral gavage. Two weeks after BSRE or MSM treatment, all rats, excluding those in the NC group, received an intra-articular injection of 3 mg of MIA in 50 µL saline into the right knee to induce osteoarthritis, as described in previous papers [45,46]. The rats in the NC group were injected with 50 μL saline solution instead of MIA solution. We conducted the measurements and sample harvesting 3 weeks post-MIA injection, specifically, on day 21, to assess the effects of the treatments. Upon conclusion of the experimental period, the rats were anesthetized with 2–3% isoflurane/N_2_O/O_2_ mixture, and the thickness of both knees was measured with a digital caliper. Blood was collected from the heart and serum was obtained from the blood by centrifugation. Subsequent to blood collection, the rats were euthanized by carbon dioxide asphyxiation, after which the knee joints and the synovium were promptly harvested for further analysis.

### 4.5. Micro-CT Analysis

To investigate changes in the knee joint micro-architecture, micro-CT analysis was conducted using micro-CT scanner and image analysis software μCT V6.1 (VivaCT 80, Scanto Medical AG, Brűttisellen, Switzerland) at the Chuncheon Center of the Korea Basic Science Institute, as described previously [46]. The femorotibial joint underwent scanning, the results of which were reconstructed into three-dimensional images for three-dimensional morphometric parameters analysis. Within the femorotibial joint, BS/BV (%) of the subchondral bone was evaluated to determine the severity of cortical bone erosion. Additionally, in the tibia, parameters such as BV/TV (%), Tb.N (1/mm), and Tb.Th (mm) of the metaphysis were analyzed to discern trabecular structural alterations.

### 4.6. Histological Examination

The knee joints were fixed in 4% paraformaldehyde, decalcified, and embedded in paraffin. Subsequently, the tissues were cut into 5 μm-thick sections and stained with H&E and Safranin O according to the manufacturer’s instructions. The stained tissues were scrutinized under a microscope (AxioImager, Carl Zeiss, Jena, Germany). The randomly selected areas were photographed at 100× magnification and blindly examined. The severity of knee osteoarthritis was assessed according to the OARSI scoring system [47].

### 4.7. IF Staining

Sections of paraffin-embedded knee joint tissues, 5 µm thick, were deparaffinized and blocked with 5% bovine serum albumin. IF staining was performed using antibodies against COL2A1 and aggrecan along with fluorochrome-conjugated secondary antibodies (Alexa-488 or 564). The nuclei were counterstained with DAPI. The stained tissues were observed under a microscope (AxioImager, Carl Zeiss), with randomly selected areas photographed at 400× magnification and examined blindly. The immune-positive cells were quantified with Image M1 Software AxioVision 4.8 (Carl Zeiss).

### 4.8. Measurement of Inflammatory Mediators, Cytokines, and MMP Levels in the Serum

The level of nitrite, as an indicator of NO, was measured in the sera using a Griess reagent system, as per the manufacturer’s instructions. The levels of PGE2, LTB4, IL-1β, TNF-α, MMP-3, MMP-9, and MMP-13 in the sera were quantified using the relevant ELISA kits in accordance with the manufacturer’s instructions.

### 4.9. Real-Time RT-PCR

Real-time RT-PCR was conducted as described previously [46]. Total RNA from the synovium of the knee joint was extracted using TRIzol reagent. Single-strand complementary DNA was synthesized using the HyperScript^TM^ RT master mix kit, and real-time PCR was conducted employing the QuantiNova SYBR Green PCR kit (Qiagen) and Rotor-Gene 3000 instrument (Corbett Research, Mortlake, Australia). The sequences of the primers used in this study are shown in Table 1. Data analysis was performed using the Rotor-Gene 6000 Series system software program, version 6 (Corbett Research). The relative mRNA expression levels of target genes were normalized to those of glyceraldehyde 3-phosphate dehydrogenase (GAPDH).

### 4.10. Statistical Analysis

All results are presented as the mean ± standard error of mean (SEM). Student’s *t*-test and one-way analysis of variance (ANOVA) were used. These analyses were conducted using Statistical Analysis System, Window version 9.4 (SAS Institute, Cary, NC, USA). *p*-value less than 0.05 was considered indicative of statistical significance.

## 5. Conclusions

This study aimed to assess the anti-osteoarthritis efficacy of BSRE (FlexiBos^®^), which contains 110 mg/g of the sum of AKBA and KBA, in SD rats with osteoarthritis induced by MIA. BSRE was orally administered at doses of 50, 75, or 100 mg/kg BW/day for 5 weeks, and osteoarthritis was induced by injecting MIA (3 mg/50 μL/rat) into the knee joint space after 2 weeks of BSRE administration. In our animal models, BSRE mitigated knee joint swelling, inhibited cartilage degradation, and enhanced COL2A1 and aggrecan expression in cartilage. BSRE at 75 and 100 mg/kg BW/day reduced serum PGE2, LTB4, IL-6, and TNF-α levels, as well as MMP-3 and -13 contents. Moreover, BSRE diminished iNOS, COX-2, 5-LOX, IL-6, and TNF-α mRNA expression in the cartilage synovium and decreased MMP-2, -3, and -13 mRNA expression. These findings suggest that BSRE exhibits anti-osteoarthritis efficacy by suppressing inflammatory responses through reducing the expression of inflammatory mediators and cytokines and by inhibiting cartilage matrix decomposition by downregulating MMP expression. The results point to the potential of BSRE as a functional raw material for enhancing joint and cartilage health, pending future human application tests.

## Figures and Tables

**Table 1:** Primer sequences used in this study.

Target Gene	Forward Primer (5′-3′)	Reverse Primer (5′-3′)
*5-Lox*	CCATCCAGCTCAACCAAACC	GATGTGTGCGGAGAAGATGG
*Cox-2*	TGCGATGCTCTT CCGAGCTGTGCT	TCAGGAAGTTCCTTATTTCCTTTC
*Il-1* *β*	CACCTCTCAAGCAGAGCACAG	GGGTTCCATGGTGAAGTCAAC
*Il-6*	TCCTACCCCAACTTCCAATGCTC	TTGGATGGTCTTGGTCCTTAGCC
*iNos*	CACCACCCTCCTTGTTCAAC	CAATCCACAACTCGCTCCAA
*Mmp-2*	TGGGGGAGATTCTCACTTTG	CCATCAGCGTTCCCATACTT
*Mmp-3*	TGGGAAGCCAGTGGAAATG	CCATGCAATGGGTAGGATGAG
*Mmp-9*	TGCTCCTGGCTCTAGGCTAC	TTGGAGGTTTTCAGGTCTCG
*Mmp-13*	TGGCGACAAAGTAGATGCTG	TGGCATGACTCTCACAATGC
*Tnf-* *α*	AAATGGGCTCCCTCTCATCAGTTC	TCTGCTTGGTGGTTTGCTACGAC
*Gapdh*	CTCAACTACATGGTCTACATGTTCCA	CTTCCCATTCTCAGCCTTGACT

## Data Availability

The data used to support this study are included with the article.
